# Leveraging TCGA gene expression data to build predictive models for cancer drug response

**DOI:** 10.1186/s12859-020-03690-4

**Published:** 2020-09-30

**Authors:** Evan A. Clayton, Toyya A. Pujol, John F. McDonald, Peng Qiu

**Affiliations:** 1 Integrated Cancer Research Center, School of Biological Sciences, Georgia Institute of Technology, Atlanta, GA USA; 2 School of Industrial and Systems Engineering, Georgia Institute of Technology, Atlanta, GA USA; 3 Department of Biomedical Engineering, Georgia Institute of Technology and Emory University, 950 Atlantic Dr NW, 30332-0230, Atlanta, GA 404-385-1656 USA

**Keywords:** Personalized oncology, Machine learning, Drug response, Predictive models

## Abstract

**Background:**

Machine learning has been utilized to predict cancer drug response from multi-omics data generated from sensitivities of cancer cell lines to different therapeutic compounds. Here, we build machine learning models using gene expression data from patients’ primary tumor tissues to predict whether a patient will respond positively or negatively to two chemotherapeutics: 5-Fluorouracil and Gemcitabine.

**Results:**

We focused on 5-Fluorouracil and Gemcitabine because based on our exclusion criteria, they provide the largest numbers of patients within TCGA. Normalized gene expression data were clustered and used as the input features for the study. We used matching clinical trial data to ascertain the response of these patients via multiple classification methods. Multiple clustering and classification methods were compared for prediction accuracy of drug response. Clara and random forest were found to be the best clustering and classification methods, respectively. The results show our models predict with up to 86% accuracy; despite the study’s limitation of sample size. We also found the genes most informative for predicting drug response were enriched in well-known cancer signaling pathways and highlighted their potential significance in chemotherapy prognosis.

**Conclusions:**

Primary tumor gene expression is a good predictor of cancer drug response. Investment in larger datasets containing both patient gene expression and drug response is needed to support future work of machine learning models. Ultimately, such predictive models may aid oncologists with making critical treatment decisions.

## Background

The goal of personalized medicine is to tailor treatments for individuals based on unique characteristics of their genetic background. Given the vast variety of cancers and the inherent molecular heterogeneity of the disease, personalized medicine in cancer can be particularly effective [1]. By studying molecular profiles of tumors, one can potentially discover biomarkers for drug sensitivity, resistance or adverse effects that may be helpful in predicting drug response [2, 3]. Recent successes demonstrated small molecule inhibitors which target pathways upregulated in cancer patients [4]. Further, breast cancer has long served as a model for successful personalized oncology, by administering treatments specific for HER2-positive patients [5].

While personalized oncology has shown signs of promise, not all cancers have such well-defined targetable pathways [6]. This has led to the recent emergence of machine learning for predicting cancer drug response. However, the success of predictive models depends largely on the availability of substantial amounts of training data. For this reason, most predictive studies to date have utilized genomic and transcriptomic profiles from panels of cancer cell lines as features for building models [7–18], as documented in a recent review [19]. This method, though encouraging, has had rather minimal success. Low interpretability and limited accuracy are the drawbacks of predicting in vivo response based on in vitro data. Further, technical challenges include high dimensionality of molecular data which is prone to overfitting and can lead to deceptive associations from intrinsically multiplex gene networks [20]. Together, these challenges have muddled attempts to build clinically relevant predictive models for drug response.

Here, we attempt to develop an improved model for predicting drug response in patients. We apply several well-established machine learning techniques to address the technical challenges. First, to reduce dimensionality we utilize optCluster [21], an R package for determining the optimal clustering algorithm and optimal number of clusters. OptCluster identifies highly similar or repetitive expression patterns from genes, and clusters them into gene modules. This method reduces the number of features while also minimizing the amount of information loss. Secondly, we predict drug response using the random forest algorithm [22] in order to protect against overfitting; a common issue with many machine learning methods. Random forest is an ensemble method which builds decision trees. This approach deters overfitting by incorporating a variety of features and leveraging a majority vote when performing classification [23].

**Fig. 1:**
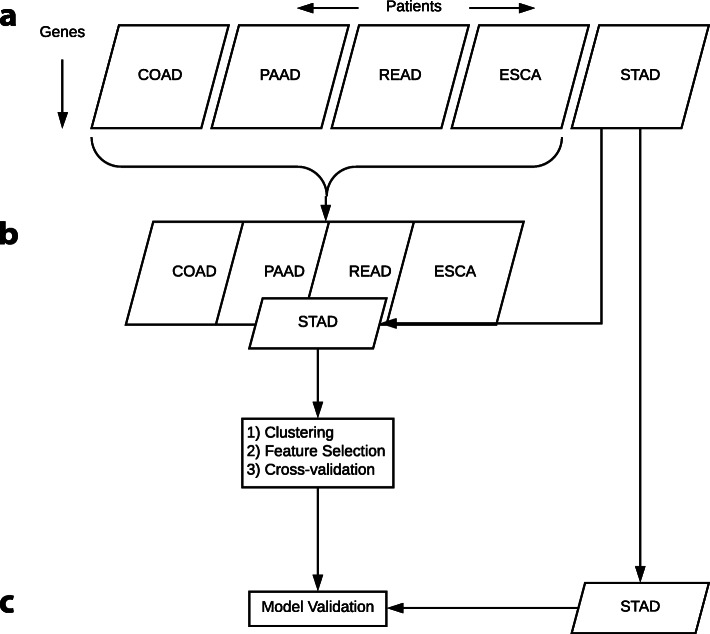
Scheme of data division throughout our study. Shown here is the workflow for training and validating the 5-FU model. The same method applies to the GCB model. **a** Gene expression data for five cancer types with 5-FU drug response data were downloaded from TCGA. **b** Three steps were performed on the training data made up from all five cancer types: clustering via OptCluster, feature selection via random forest and prediction via random forest with cross-validation to train the model. **c** Model validation was performed on half of the most populous cancer type (STAD) held out as an independent validation data set

The utility of drug response models built from in vitro data is often limited, because of the genetic and environmental differences between cell lines and patient-derived samples. Not until recently, has in vivo data been publicly available for research. For the first time, an extensive compendium of pan-cancer datasets has been released by The Cancer Genome Atlas (TCGA), which provide both clinical drug response data and gene expression profiles from primary tumor patient samples spanning multiple cancer types [24]. In this study, we take a novel approach to build predictive models (Fig. 1) for drug response by utilizing this in vivo data. We observe robust prediction and we evaluate predictive gene modules that are implicated in biological pathways critical to drug response.

## Results

### Drug selection results

Our study is based on data obtained from the National Cancer Institute’s TCGA database [24]. TCGA provides both patients’ clinical trial data and transcriptomic data from patients’ primary tumor samples. This data includes expression levels for 60,483 genes including protein-coding genes, non-coding RNA genes and pseudogenes. We used the Genomic Data Commons API to download the Upper-Quartile Normalized Fragments per Kilobase per Million mapped reads (UQ-FPKMs) from the patients’ primary tumor samples. The clinical trial data consisted of 12,051 records with data for 32 cancer types. Each record contains clinical trial data for an individual patient. There are multiple clinical trials in the database and a patient has one record for each clinical trial of which they participated (i.e. a patient can have two records if they participated in different trials). Each record includes information about: drugs administered, patient demographics, temporal data of the study, and response of the patient. For the purposes of classification, we define a responder as a patient who had partial or complete response and a non-responder as a patient who had a clinical progressive disease or stable disease response. One pan-cancer model for each drug was created by including all the cancer types that the selected drug had treated. Only patients on single drug therapy throughout the entire duration of treatment were retained in the study. Based on these criteria, Fluorouracil (5-FU) and Gemcitabine (GCB) were chosen because they provided the highest number of records. Our study included two models: (1) 5-FU pan-cancer and (2) GCB pan-cancer. See Table 1 for the counts of each model.

**Table 1:** Patient counts for each model by response

Model	Responder Count	Non-responder Count	Total Count
Fluorouracil pan-cancer^a^	34	24	58
Gemcitabine pan-cancer^b^	37	55	92

^a^Fluorouracil pan-cancer includes: colon adenocarcinoma, esophageal carcinoma, pancreatic adenocarcinoma, rectum adenocarcinoma, stomach adenocarcinoma

^b^Gemcitabine pan-cancer includes: bladder urothelial carcinoma, breast invasive carcinoma, cervical squamous cell carcinoma and endocervical adenocarcinoma, cholangiocarcinoma, head and neck squamous cell carcinoma, liver hepatocellular carcinoma, lung adenocarcinoma, ovarian serous cystadenocarcinoma, pancreatic adenocarcinoma, pheochromocytoma and paraganglioma, sarcoma, skin cutaneous melanoma, testicular germ cell tumors, uterine corpus endometrial carcinoma

### OptCluster results

**Fig. 2:**
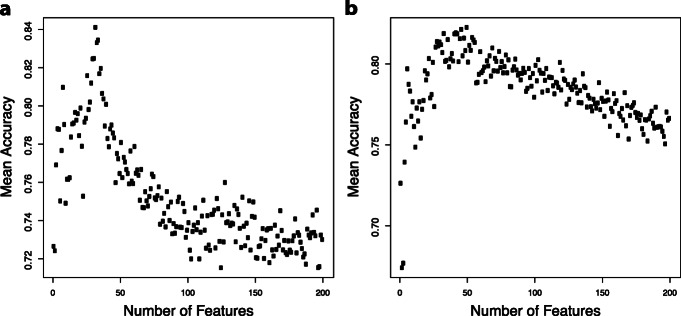
Accuracy of random forest by number of clusters (using clara clustering algorithm). **a** Mean accuracy (200x cross-validation) was calculated using 1–204 clusters in order of importance from the 5-FU pan-cancer model. **b** Mean accuracy (200x cross-validation) was calculated using 1–192 clusters in order of importance from the GCB pan-cancer model

We report the results of the most accurate clustering algorithm from optCluster in Table 2. Clara coupled with several classification algorithms provided the best gene modules with a cross-validation mean accuracy of 84.1% (sd:10.7%) for 5-FU and 82.3% (sd:8.6%) for GCB. In Fig. 2, we see how accuracy changes relative to the number of selected clusters. The peak accuracy was with 32 and 50 clusters for 5-FU and GCB, respectively. We tested other classification methods, support vector machines and logistic regression, but both yielded worse results. Cross-validation accuracy for support vector machine was 81% for 5-FU and 71.5% for GCB and logistic regression was 77.0% for 5-FU and 73.0% for GCB. There was minimal impact on accuracy when including demographic data of the patients (gender, age, cancer type and cancer stage) [5-FU: 83.6%; Gemcitabine: 79.1%]. Prediction accuracies from the cross-validation analysis of both drugs, as well as validation on a separately held-out dataset, are summarized in Table 2.

**Table 2:** Number of clusters and mean accuracy for pan-cancer models

	Fluorouracil			Gemcitabine		
Clustering Method	# Considered Clusters^b^	# Selected Clusters^c^	Average Accuracy	# Considered Clusters^b^	# Selected Clusters^c^	Average Accuracy
Optcluster (Clara) with RF^a^	204	32	84.1%	192	50	82.3%
Optcluster (Clara) with SVM^a^	204	32	81.0%	192	50	71.5%
Optcluster (Clara) with logistic regression	204	32	77.0%	192	50	73.0%
Optcluster (Clara) with RF and demographics	204	32	83.6%	192	50	79.1%
Model Validation (Clara)	204	32	52.9%	192	50	82.1%
Model Validation (Clara with Tuning)	204	32	52.9%	192	50	85.7%

^a^RF is for random forest; SVM is for support vector machine

^b^Number of considered clusters represents the number of clusters entered into random forest for variable importance ranking

^c^Number of selected clusters is number of clusters selected by random forest for classification

**Fig. 3:**
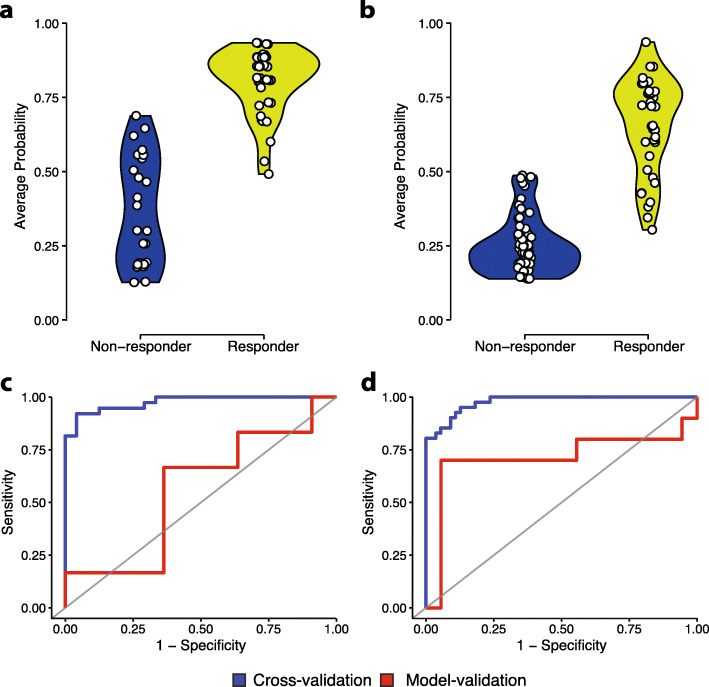
Random forest classifier performance for pan-cancer models. **a** Comparison of the cross-validation predicted probabilities between non-responders and responders for the 5-FU pan-cancer model. **b** Comparison of the cross-validation predicted probabilities between non-responders and responders for the GCB pan-cancer model. **c** ROC curve for 5-FU pan-cancer model; Cross-validation (Sensitivity: 0.97 Specificity: 0.66 AUC: 0.98) Model-validation (Sensitivity: 0.64 Specificity: 0.33 AUC: 0.56). **d** ROC curve for GCB pan-cancer model; Cross-validation (Sensitivity: 0.80 Specificity: 1.0 AUC: 0.98) Model-validation (Sensitivity: 0.70 Specificity: 0.94 AUC: 0.71)

In Fig. 3a-b, the cross-validation predicted probabilities for non-responders and responders are plotted. The 5-FU model is particularly strong at identifying responders (97% sensitivity) while the GCB model is better at classifying non-responders (100% specificity).

### Model validation & ROC curves

The ROC curves in Fig. 3c-d demonstrate the sensitivity and specificity of the cross-validated model based on training data and additional validation based on test data. The prediction performance on the test data showed an increase in the accuracy for GCB by 3 percentage points to 85.7%. We did not see the same improvement in 5-FU, which dropped to 52.9% accuracy. The training data for both models performed with AUC = 0.98. More interestingly, we see the GCB validation curve still classifies well with AUC = 0.71, while the 5-FU validation curve is barely above the random classifier line; showing it doesn’t perform much better than chance. This decrease in accuracy may be attributed to sample size or difficulty predicting on the cancer type (stomach adenocarcinoma) used for this validation.

### Gene set enrichment analysis

**Fig. 4:**
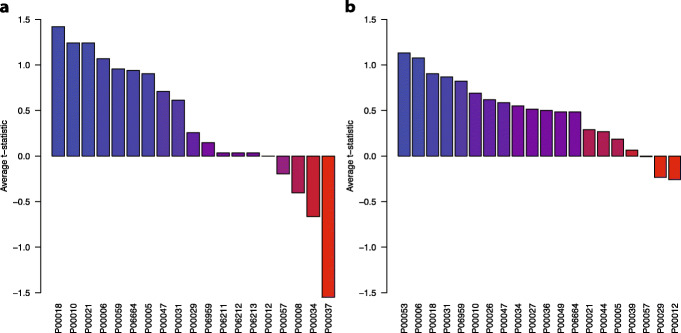
Average t-statistics for PANTHER pathways enriched in final models. Plots show the average t-statistic for most prevalent pathways. Mean gene expression values for each gene in a given pathway were compared for non-responders vs responders. **a** 309 identified genes from 5-FU pan-cancer model across pathways with highest gene percentage. **b** 1158 identified genes from GCB pan-cancer model across pathways with highest gene percentage

Once we established an optimal model for predicting drug response, we performed a gene set enrichment analysis using PANTHER [25]. We analyzed the genes within each of the clara gene modules (S1 Table, Additional file 1). The significantly enriched biological pathways for Gemcitabine are Inflammation mediated by chemokine and cytokine signaling pathway (P = < 9.3 × 10–4), T cell activation (*P* = 2.4 × 10–5) and B cell activation (*P* = 9.9 × 10–5). For a list of the top 20 pathways based on the percentage of genes within that pathway refer to the S2 Table, Additional file 1. In addition, Fig. 4 shows the relationship of gene expression level between responders and non-responders for each of these pathways. A high positive number, such as in P00018 indicates that the mean gene expression for the responders in pathway, P00018, are higher than that of the non-responders. The opposite holds true for the negative values.

## Discussion

In our study we have shown that primary tumor gene expression can be a good predictor of cancer drug response. By utilizing different clustering and classification methods we predicted cancer drug response with validation accuracy of up to 86%.

Our analyses show the GCB model performs stronger than the 5-FU model (Table 2). We attribute the GCB model’s high prediction accuracy to multiple facets. First, the GCB model had a more substantial sample size of 92 patients. This was undoubtedly beneficial, as the algorithm was able to take advantage of the increased diversity in the training data to build a rigorous model that was able to successfully predict on a new dataset. Secondly, when clustering gene expression levels with a similarity threshold, the GCB data was grouped into fewer clusters; an indication the dataset was more homogenous and better-suited for dimension reduction. Further, random forest cross-validation accuracy was optimized at a higher number of clusters than 5-FU (73% more genes), leading us to believe the larger sample size of GCB helped the model to better differentiate informative features from noise. Another important variation between models is the specific cancer type on which validation performance was evaluated. Our 5-FU training model was only able to predict with 76% accuracy on STAD patients (see S3 Table, Additional file 1) compared to the 84.1% overall accuracy. This provides evidence that the pan-cancer model was better at predicting drug response for certain cancer types.

We infer from our results that some drugs target mechanisms that are shared across most cancers, while others may target mechanisms specific to certain cancer families. Our GCB pan-cancer model predicts all cancer types at comparable levels to that of the overall accuracy. On the other hand, a much higher variation in accuracy is seen from the 5-FU model (S3 Table, Additional file 1). In the cases where the targeted mechanisms of a drug are different across cancer families, we would expect to see a reduction in the prediction accuracies of cancers with dissimilar mechanisms. When more data becomes available, future work can test the performance of models built on molecularly similar cancers of the same histology or anatomy, as suggested by a recent study [26].

While biologically informed models have been shown to be successful [27–30], we decided to infer the biology from our empirically-derived model using gene set enrichment analysis. Our analysis reveals that many biological pathways relevant to drug metabolism and cancer are present in the most predictive gene modules. Several pathways were found in both 5-FU and GCB pan-cancer models. 7.1% and 3.3% of genes in the Integrin signaling pathway were present in 5-FU and GCB models, respectively (S2 Table, Additional file 1). Integrins are adhesive receptors that allow cells to respond to signals from the surrounding microenvironment by interacting with extracellular matrix [31]. They have been implicated in cell adhesion-mediated drug resistance, a pro-survival and anti-apoptotic function [32]. A second pathway in common is the WNT signaling pathway, which has 11.8% and 5.7% of its genes found in 5-FU and GCB models, respectively (S2 Table, Additional file 1). Previous research has linked this pathway to tumorigenesis via cell fate determination and cell migration [33]. Moreover, upregulation of the WNT pathway is involved in more than 30% of gastric cancer cases [34]. Stomach adenocarcinoma is the most populous cancer type in the 5-FU pan-cancer data (Table 1), potentially driving this model’s selection of gastric cancer associated genes. The GCB pan-cancer model is significantly enriched for genes from the “inflammation mediated by chemokine and cytokine signaling” pathway (*P* = 9.3 × 10–4). Chemokines direct trafficking and migration of immune cells and inhibition of these proteins has been proven effective in preventing the accumulation of leukocytes near sites of inflammation [35]. We believe the presence of these pathways in our models provides insight into their biological relevance and tactics for predicting cancer drug response.

## Conclusions

The results of our final approach for prediction cancer drug responses were conclusively accurate and, more importantly, interpretable. Our classifier selected genes that are integral parts of drug metabolism and cancer biology. This combination of accuracy and interpretability has been difficult to achieve in predictive models attempted in the past. We attribute our success to the utilization of in-vivo gene expression data, which eliminates the need to extrapolate human drug responses from cell line or other in-vitro features. Furthermore, our implementation of optCluster and random forest provided us with a method to perform dimension reduction in a biologically informative manner. Feature ranking, as we have shown, selects biologically relevant genes, that may yield new therapeutic targets. While recent discussion has suggested machine learning can appear as “alchemy” [36], we encourage the continued effort in the field of personalized cancer medicine as it bears great potential for benefiting patients. To conclude, predicting cancer drug response from patient RNA-seq data will be an important tool for personalized oncology. We anticipate that predictive models, such as the ones we present, will continue to grow more powerful and will aid clinicians and patients in selecting first or second-line therapies.

## Methods

### Clustering & Variable Selection

We focused our study on pan-cancer models. Pan-cancer models were shown, by a previous study [14], to out-perform single cancer models. Further, we performed our own empirical analysis with similar results strengthening this choice (see Supplementary Methods, Additional file 1). Next, we implemented clustering (i.e. grouping gene) to reduce the number of dimensions. We evaluated six clustering algorithms (clara, hierarchical, k-means, model, pam, and sota) [37–41] using the top 5000 genes, based on highest gene expression variability (S1 Figure, Additional file 1). Then we chose the clustering algorithm that provided the highest percentage of correct predictions (prediction accuracy).

To determine accuracy, we used the mean value for each gene cluster, hereunto gene module, as a feature for prediction. The number of observations (58 for 5-FU and 92 for GCB) was much lower than the chosen number of clusters (~ 200), so feature selection was performed. We used the machine learning technique, random forest, to determine feature importance. Random forest utilizes a forest of decision trees to split the data into multiple subsets based on predictive power. To stabilize the ranked list of feature importance, we took the mean Gini value for each gene module after 200 random forest runs. The mean decrease of Gini index was selected because it is the recommended performance metric for imbalanced data [42].

To find the best clustering algorithm for our study, we performed clustering and classification in a five-step process (S2 Figure, Additional file 1). We used the R package, optCluster, and relied on internal validation to determine the stability of the clusters [21].

1. The algorithm was run with N number of clusters (where N is in the range of 180–220; accuracies were observed to drop outside this cluster number range).
2. Random forest was used to determine feature importance and perform feature selection.
3. For each top n most important features (where n is between 1 to N), random forest was used to classify each patient.
4. The highest classification method was logged for the best value of N and n for each clustering method and for each drug model.
5. The best model from the previous step was selected and the parameters were tuned to capture any additional accuracy.

We completed a comprehensive analysis by trying other classification methods and including other types of data. We compared the accuracy obtained from random forest to the that of logistic regression and support vector machines using the optimal random forest features. We also tested if the model would improve by including demographic data of the patients (gender, age, cancer type and cancer stage) on the accuracy of the model. All approaches yielded worse results.

### Model validation

The final 5-FU and GCB models were trained on their respective pan-cancer data in which half of the patients from the most populous cancer, STAD or PAAD respectively, were held out as validation sets (Fig. 1). To reduce the impact of small sample size in the pan-cancer models, we used bootstrapping as an up-sampling method to increase the size of the training set by 50%. The up-sampled training set consisted of: (1) all the original samples and (2) a random sample selected from the original training set. The random sample is half the size of the original training set and generated by sampling with replacement. The two validation sets consisted of 17 randomly selected STAD patients and 28 randomly selected PAAD patients for the 5-FU and GCB models respectively. We validated our selected models by calculating the prediction accuracy on the independent validation sets comprised from 50% of the most populous cancer type.

### Statistical methods: gene enrichment analysis

Our hypothesis stated the selected genes in our final model would be enriched for pathways involved in drug metabolism or cell signaling. To test this hypothesis, we performed two statistical tests. First, we performed a PANTHER overrepresentation test. Using the results of the PANTHER analysis, we performed a t-test comparing the mean gene expression of responders and non-responders for the top twenty pathways based on the percent of genes hit against the total number of genes in the pathway (See Table S2, Additional file 1).

## Supplementary information

**Additional file 1:** **Figure S1.** Dimension reduction of genes. **Figure S2.** Survival data as a predictor of drug response. **Table S1.** Genes selected by random forest variable importance. **Table S2.** Top 20 PANTHER pathways in models by gene percent. **Table S3.** Accuracy by cancer type. **Supplementary Methods** Data pre-processing, gene standardization and gene selection

## Data Availability

The source code and data associated with the manuscript are available in the cancer_drug_response repository: https://github.com/clayton0171/cancer_drug_response

## Abbreviations

5-FU: 5-Fluorouracil
GCB: Gemcitabine
PAAD: Pancreatic adenocarcinoma
STAD: Stomach adenocarcinoma
