# Supplementary material for: Leveraging TCGA gene expression data to build predictive models for cancer drug response
Source: BMC Bioinformatics. 2020 Sep 30;21(Suppl 14):364. doi: 10.1186/s12859-020-03690-4 (PMC7526215; doi:10.1186/s12859-020-03690-4)
Supplement: Supplementary file 1 — Additional file 1: Figure S1. Dimension reduction of genes. Figure S2. Survival data as a predictor of drug response. Table S1. Genes selected by random forest variable importance. Table S2. Top 20 PANTHER pathways in models by gene percent. Table S3. Accuracy by cancer type. Supplementary Methods Data pre-processing, gene standardization and gene selection [file 12859_2020_3690_MOESM1_ESM.docx]

# **S1 Figure: Dimension reduction of genes**


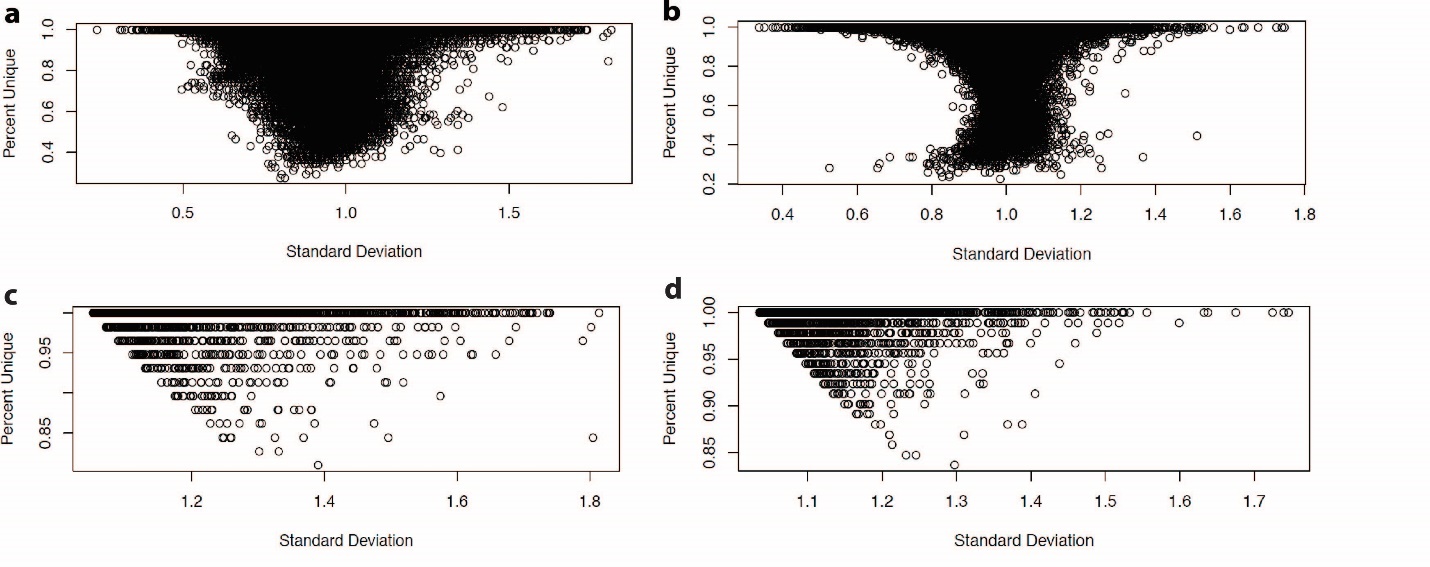


**S1 Figure: Dimension reduction of genes.** Figures of the 5,000 selected gene based on gene expression variability. 2,500 and 7,500 genes were also tried but yielded worse results, and hence not used for the study. **(a)** Percent unique gene expression values vs standard deviation for 5-FU pan cancer model **(b)** Percent unique gene expression values vs standard deviation for GCB pan cancer model **(c)** Percent unique gene expression values vs standard deviation for top 5,000 5-FU pan cancer model **(d)** Percent unique gene expression values vs standard deviation for top 5,000 GCB pan cancer model

# **S2 Figure: Survival data as a predictor of drug response**

# **
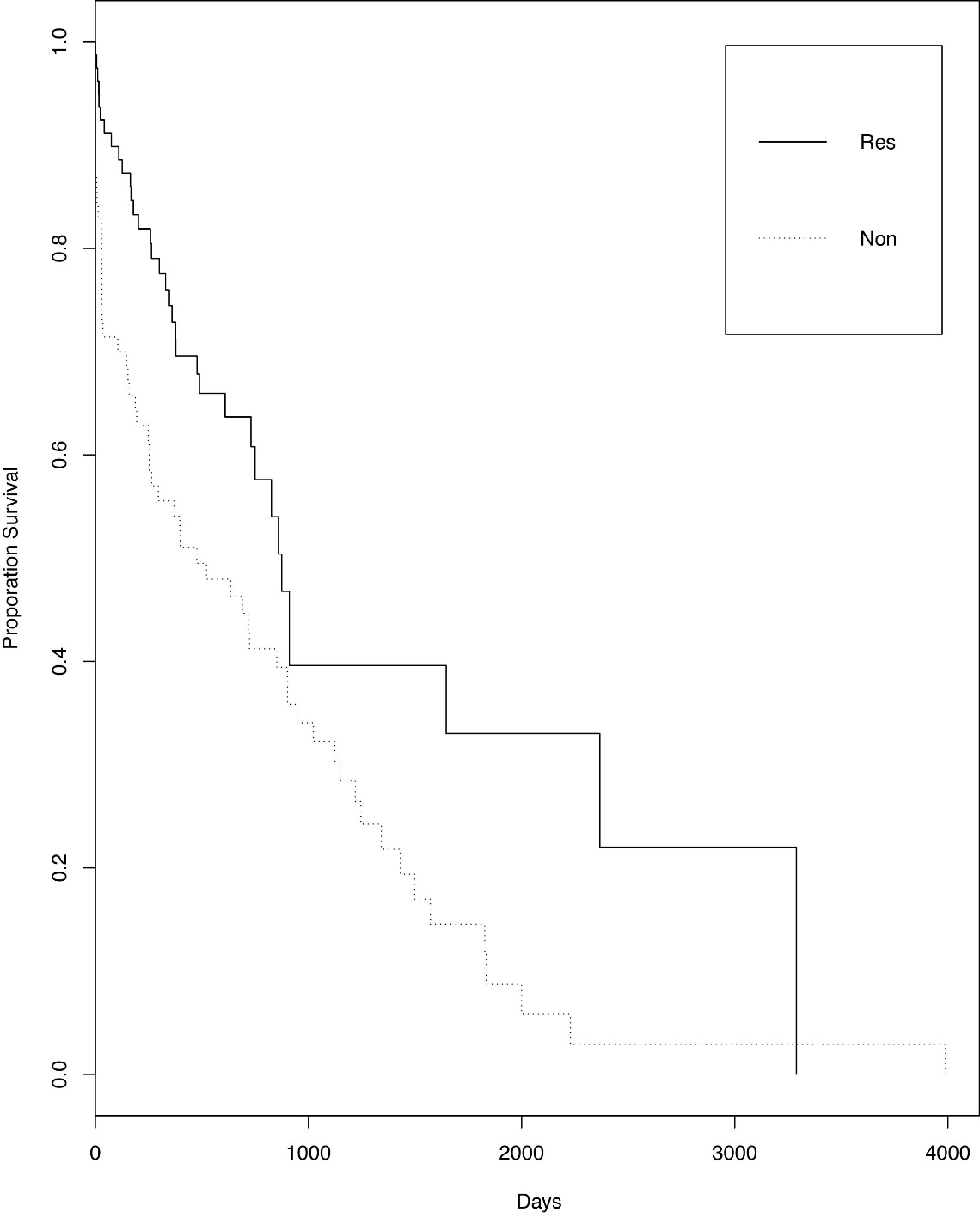
**

**S2 Figure: Survival data as a predictor of drug response.** Survival data was downloaded for all patients from both GCB and 5FU models. Kaplan–Meier plot for responders (res) and non-responders (non) depicting patient survival over time.

# **S1 Table: Genes selected by random forest variable importance**

| **Gemcitabine Panther Gene List** | |  |
| --- | --- | --- |
| **Ensemble gene ID** | **Pathway ID** | **Pathway Name** |
| ENSG00000137275 | P00006 | Apoptosis signaling pathway |
| ENSG00000169413 | P00005 | Angiogenesis |
| ENSG00000067900 | P00016 | Cytoskeletal regulation by Rho GTPase |
| ENSG00000067900 | P00031 | Inflammation mediated by chemokine and cytokine signaling pathway |
| ENSG00000067900 | P06959 | CCKR signaling map |
| ENSG00000187764 | P00007 | Axon guidance mediated by semaphorins |
| ENSG00000101144 | P00052 | TGF-beta signaling pathway |
| ENSG00000101144 | P06664 | Gonadotropin releasing hormone receptor pathway |
| ENSG00000079215 | P00037 | Ionotropic glutamate receptor pathway |
| ENSG00000079215 | P00039 | Metabotropic glutamate receptor group III pathway |
| ENSG00000130821 | P00043 | Muscarinic acetylcholine receptor 2 and 4 signaling pathway |
| ENSG00000130821 | P00044 | Nicotinic acetylcholine receptor signaling pathway |
| ENSG00000080503 | P00057 | Wnt signaling pathway |
| ENSG00000099956 | P00057 | Wnt signaling pathway |
| ENSG00000275837 | P00057 | Wnt signaling pathway |
| ENSG00000073584 | P00057 | Wnt signaling pathway |
| ENSG00000132639 | P00001 | Adrenaline and noradrenaline biosynthesis |
| ENSG00000132639 | P00002 | Alpha adrenergic receptor signaling pathway |
| ENSG00000132639 | P00037 | Ionotropic glutamate receptor pathway |
| ENSG00000132639 | P00039 | Metabotropic glutamate receptor group III pathway |
| ENSG00000132639 | P00040 | Metabotropic glutamate receptor group II pathway |
| ENSG00000132639 | P00042 | Muscarinic acetylcholine receptor 1 and 3 signaling pathway |
| ENSG00000132639 | P00043 | Muscarinic acetylcholine receptor 2 and 4 signaling pathway |
| ENSG00000132639 | P00044 | Nicotinic acetylcholine receptor signaling pathway |
| ENSG00000132639 | P04373 | 5HT1 type receptor mediated signaling pathway |
| ENSG00000132639 | P04374 | 5HT2 type receptor mediated signaling pathway |
| ENSG00000132639 | P04375 | 5HT3 type receptor mediated signaling pathway |
| ENSG00000132639 | P04376 | 5HT4 type receptor mediated signaling pathway |
| ENSG00000132639 | P04377 | Beta1 adrenergic receptor signaling pathway |
| ENSG00000132639 | P04378 | Beta2 adrenergic receptor signaling pathway |
| ENSG00000132639 | P04379 | Beta3 adrenergic receptor signaling pathway |
| ENSG00000132639 | P04380 | Cortocotropin releasing factor receptor signaling pathway |
| ENSG00000132639 | P04391 | Oxytocin receptor mediated signaling pathway |
| ENSG00000132639 | P04394 | Thyrotropin-releasing hormone receptor signaling pathway |
| ENSG00000132639 | P05734 | Synaptic_vesicle_trafficking |
| ENSG00000132639 | P05912 | Dopamine receptor mediated signaling pathway |
| ENSG00000132639 | P05915 | Opioid proenkephalin pathway |
| ENSG00000132639 | P05916 | Opioid prodynorphin pathway |
| ENSG00000132639 | P05917 | Opioid proopiomelanocortin pathway |
| ENSG00000057252 | P02727 | Androgen/estrogene/progesterone biosynthesis |
| ENSG00000167780 | P02727 | Androgen/estrogene/progesterone biosynthesis |
| ENSG00000100485 | P00005 | Angiogenesis |
| ENSG00000100485 | P00010 | B cell activation |
| ENSG00000100485 | P00018 | EGF receptor signaling pathway |
| ENSG00000100485 | P00021 | FGF signaling pathway |
| ENSG00000100485 | P00032 | Insulin/IGF pathway-mitogen activated protein kinase kinase/MAP kinase cascade |
| ENSG00000100485 | P00034 | Integrin signalling pathway |
| ENSG00000100485 | P00036 | Interleukin signaling pathway |
| ENSG00000100485 | P00047 | PDGF signaling pathway |
| ENSG00000100485 | P00048 | PI3 kinase pathway |
| ENSG00000100485 | P00053 | T cell activation |
| ENSG00000100485 | P04393 | Ras Pathway |
| ENSG00000100485 | P06664 | Gonadotropin releasing hormone receptor pathway |
| ENSG00000066336 | P00036 | Interleukin signaling pathway |
| ENSG00000104549 | P00014 | Cholesterol biosynthesis |
| ENSG00000010671 | P00010 | B cell activation |
| ENSG00000278195 | P00026 | Heterotrimeric G-protein signaling pathway-Gi alpha and Gs alpha |
| ENSG00000278195 | P00027 | Heterotrimeric G-protein signaling pathway-Gq alpha and Go alpha |
| ENSG00000138378 | P00018 | EGF receptor signaling pathway |
| ENSG00000138378 | P00036 | Interleukin signaling pathway |
| ENSG00000138378 | P00038 | JAK/STAT signaling pathway |
| ENSG00000138378 | P00047 | PDGF signaling pathway |
| ENSG00000126561 | P00018 | EGF receptor signaling pathway |
| ENSG00000126561 | P00036 | Interleukin signaling pathway |
| ENSG00000126561 | P00038 | JAK/STAT signaling pathway |
| ENSG00000126561 | P00047 | PDGF signaling pathway |
| ENSG00000079950 | P00001 | Adrenaline and noradrenaline biosynthesis |
| ENSG00000079950 | P00049 | Parkinson disease |
| ENSG00000196628 | P06959 | CCKR signaling map |
| ENSG00000148737 | P00004 | Alzheimer disease-presenilin pathway |
| ENSG00000148737 | P00005 | Angiogenesis |
| ENSG00000148737 | P00012 | Cadherin signaling pathway |
| ENSG00000148737 | P00057 | Wnt signaling pathway |
| ENSG00000148516 | P06664 | Gonadotropin releasing hormone receptor pathway |
| ENSG00000028137 | P00006 | Apoptosis signaling pathway |
| ENSG00000133107 | P00004 | Alzheimer disease-presenilin pathway |
| ENSG00000105397 | P00031 | Inflammation mediated by chemokine and cytokine signaling pathway |
| ENSG00000170142 | P00060 | Ubiquitin proteasome pathway |
| ENSG00000154277 | P00049 | Parkinson disease |
| ENSG00000141968 | P00010 | B cell activation |
| ENSG00000141968 | P00031 | Inflammation mediated by chemokine and cytokine signaling pathway |
| ENSG00000141968 | P00047 | PDGF signaling pathway |
| ENSG00000141968 | P00053 | T cell activation |
| ENSG00000015285 | P00053 | T cell activation |
| ENSG00000106299 | P00016 | Cytoskeletal regulation by Rho GTPase |
| ENSG00000106299 | P00029 | Huntington disease |
| ENSG00000115085 | P00053 | T cell activation |
| ENSG00000171700 | P00026 | Heterotrimeric G-protein signaling pathway-Gi alpha and Gs alpha |
| ENSG00000171700 | P00027 | Heterotrimeric G-protein signaling pathway-Gq alpha and Go alpha |
| ENSG00000136286 | P00044 | Nicotinic acetylcholine receptor signaling pathway |
| ENSG00000067191 | P00003 | Alzheimer disease-amyloid secretase pathway |
| ENSG00000067191 | P00039 | Metabotropic glutamate receptor group III pathway |
| ENSG00000067191 | P00040 | Metabotropic glutamate receptor group II pathway |
| ENSG00000067191 | P00044 | Nicotinic acetylcholine receptor signaling pathway |
| ENSG00000067191 | P04374 | 5HT2 type receptor mediated signaling pathway |
| ENSG00000067191 | P04377 | Beta1 adrenergic receptor signaling pathway |
| ENSG00000067191 | P04378 | Beta2 adrenergic receptor signaling pathway |
| ENSG00000067191 | P04391 | Oxytocin receptor mediated signaling pathway |
| ENSG00000067191 | P04394 | Thyrotropin-releasing hormone receptor signaling pathway |
| ENSG00000110031 | P00005 | Angiogenesis |
| ENSG00000110031 | P00056 | VEGF signaling pathway |
| ENSG00000123329 | P00047 | PDGF signaling pathway |
| ENSG00000008516 | P00004 | Alzheimer disease-presenilin pathway |
| ENSG00000152689 | P00027 | Heterotrimeric G-protein signaling pathway-Gq alpha and Go alpha |
| ENSG00000149260 | P00029 | Huntington disease |
| ENSG00000170075 | P00049 | Parkinson disease |
| ENSG00000118514 | P04372 | 5-Hydroxytryptamine degredation |
| ENSG00000164674 | P05734 | Synaptic_vesicle_trafficking |
| ENSG00000196664 | P00054 | Toll receptor signaling pathway |
| ENSG00000174123 | P00054 | Toll receptor signaling pathway |
| ENSG00000166664 | P00003 | Alzheimer disease-amyloid secretase pathway |
| ENSG00000166664 | P00044 | Nicotinic acetylcholine receptor signaling pathway |
| ENSG00000275917 | P00003 | Alzheimer disease-amyloid secretase pathway |
| ENSG00000275917 | P00044 | Nicotinic acetylcholine receptor signaling pathway |
| ENSG00000118971 | P00013 | Cell cycle |
| ENSG00000118971 | P00048 | PI3 kinase pathway |
| ENSG00000112576 | P00013 | Cell cycle |
| ENSG00000163823 | P00031 | Inflammation mediated by chemokine and cytokine signaling pathway |
| ENSG00000121807 | P00031 | Inflammation mediated by chemokine and cytokine signaling pathway |
| ENSG00000183813 | P00031 | Inflammation mediated by chemokine and cytokine signaling pathway |
| ENSG00000112486 | P00031 | Inflammation mediated by chemokine and cytokine signaling pathway |
| ENSG00000173585 | P00031 | Inflammation mediated by chemokine and cytokine signaling pathway |
| ENSG00000100024 | P02771 | Pyrimidine Metabolism |
| ENSG00000012124 | P00010 | B cell activation |
| ENSG00000178562 | P00053 | T cell activation |
| ENSG00000172215 | P00031 | Inflammation mediated by chemokine and cytokine signaling pathway |
| ENSG00000004468 | P06959 | CCKR signaling map |
| ENSG00000167286 | P00053 | T cell activation |
| ENSG00000198851 | P00053 | T cell activation |
| ENSG00000160654 | P00053 | T cell activation |
| ENSG00000198821 | P00053 | T cell activation |
| ENSG00000198373 | P00060 | Ubiquitin proteasome pathway |
| ENSG00000019582 | P00053 | T cell activation |
| ENSG00000166225 | P00005 | Angiogenesis |
| ENSG00000166225 | P00021 | FGF signaling pathway |
| ENSG00000105369 | P00010 | B cell activation |
| ENSG00000007312 | P00010 | B cell activation |
| ENSG00000062598 | P00034 | Integrin signalling pathway |
| ENSG00000160219 | P00018 | EGF receptor signaling pathway |
| ENSG00000140044 | P00006 | Apoptosis signaling pathway |
| ENSG00000113361 | P00012 | Cadherin signaling pathway |
| ENSG00000113361 | P00057 | Wnt signaling pathway |
| ENSG00000144837 | P05726 | 2-arachidonoylglycerol biosynthesis |
| ENSG00000100568 | P00001 | Adrenaline and noradrenaline biosynthesis |
| ENSG00000198001 | P00054 | Toll receptor signaling pathway |
| ENSG00000099365 | P00039 | Metabotropic glutamate receptor group III pathway |
| ENSG00000099365 | P00040 | Metabotropic glutamate receptor group II pathway |
| ENSG00000099365 | P00042 | Muscarinic acetylcholine receptor 1 and 3 signaling pathway |
| ENSG00000099365 | P00043 | Muscarinic acetylcholine receptor 2 and 4 signaling pathway |
| ENSG00000099365 | P00044 | Nicotinic acetylcholine receptor signaling pathway |
| ENSG00000099365 | P05734 | Synaptic_vesicle_trafficking |
| ENSG00000213658 | P00053 | T cell activation |
| ENSG00000104951 | P04372 | 5-Hydroxytryptamine degredation |
| ENSG00000132718 | P05734 | Synaptic_vesicle_trafficking |
| ENSG00000114737 | P00031 | Inflammation mediated by chemokine and cytokine signaling pathway |
| ENSG00000114737 | P00035 | Interferon-gamma signaling pathway |
| ENSG00000196405 | P00016 | Cytoskeletal regulation by Rho GTPase |
| ENSG00000189007 | P02739 | De novo pyrimidine deoxyribonucleotide biosynthesis |
| ENSG00000189007 | P02774 | Salvage pyrimidine deoxyribonucleotides |
| ENSG00000189007 | P02775 | Salvage pyrimidine ribonucleotides |
| ENSG00000137171 | P00003 | Alzheimer disease-amyloid secretase pathway |
| ENSG00000136280 | P00018 | EGF receptor signaling pathway |
| ENSG00000197410 | P00012 | Cadherin signaling pathway |
| ENSG00000197410 | P00057 | Wnt signaling pathway |
| ENSG00000284227 | P00012 | Cadherin signaling pathway |
| ENSG00000284227 | P00057 | Wnt signaling pathway |
| ENSG00000165323 | P00012 | Cadherin signaling pathway |
| ENSG00000165323 | P00057 | Wnt signaling pathway |
| ENSG00000282908 | P00012 | Cadherin signaling pathway |
| ENSG00000282908 | P00057 | Wnt signaling pathway |
| ENSG00000169118 | P00049 | Parkinson disease |
| ENSG00000169118 | P00057 | Wnt signaling pathway |
| ENSG00000146094 | P00005 | Angiogenesis |
| ENSG00000278259 | P00044 | Nicotinic acetylcholine receptor signaling pathway |
| ENSG00000278372 | P00044 | Nicotinic acetylcholine receptor signaling pathway |
| ENSG00000128271 | P00026 | Heterotrimeric G-protein signaling pathway-Gi alpha and Gs alpha |
| ENSG00000128271 | P00027 | Heterotrimeric G-protein signaling pathway-Gq alpha and Go alpha |
| ENSG00000123454 | P00001 | Adrenaline and noradrenaline biosynthesis |
| ENSG00000123454 | P05912 | Dopamine receptor mediated signaling pathway |
| ENSG00000276231 | P00018 | EGF receptor signaling pathway |
| ENSG00000276231 | P00019 | Endothelin signaling pathway |
| ENSG00000120907 | P00002 | Alpha adrenergic receptor signaling pathway |
| ENSG00000120907 | P00026 | Heterotrimeric G-protein signaling pathway-Gi alpha and Gs alpha |
| ENSG00000150594 | P00002 | Alpha adrenergic receptor signaling pathway |
| ENSG00000150594 | P00026 | Heterotrimeric G-protein signaling pathway-Gi alpha and Gs alpha |
| ENSG00000180096 | P00049 | Parkinson disease |
| ENSG00000118363 | P04395 | Vasopressin synthesis |
| ENSG00000239900 | P02738 | De novo purine biosynthesis |
| ENSG00000114841 | P00029 | Huntington disease |
| ENSG00000140795 | P00016 | Cytoskeletal regulation by Rho GTPase |
| ENSG00000140795 | P00031 | Inflammation mediated by chemokine and cytokine signaling pathway |
| ENSG00000172667 | P00059 | p53 pathway |
| ENSG00000184845 | P00026 | Heterotrimeric G-protein signaling pathway-Gi alpha and Gs alpha |
| ENSG00000184845 | P00027 | Heterotrimeric G-protein signaling pathway-Gq alpha and Go alpha |
| ENSG00000184845 | P05912 | Dopamine receptor mediated signaling pathway |
| ENSG00000158050 | P00046 | Oxidative stress response |
| ENSG00000128951 | P02739 | De novo pyrimidine deoxyribonucleotide biosynthesis |
| ENSG00000161202 | P00004 | Alzheimer disease-presenilin pathway |
| ENSG00000161202 | P00005 | Angiogenesis |
| ENSG00000161202 | P00057 | Wnt signaling pathway |
| ENSG00000124126 | P00031 | Inflammation mediated by chemokine and cytokine signaling pathway |
| ENSG00000111674 | P00024 | Glycolysis |
| ENSG00000088367 | P05912 | Dopamine receptor mediated signaling pathway |
| ENSG00000088367 | P06587 | Nicotine pharmacodynamics pathway |
| ENSG00000181104 | P00005 | Angiogenesis |
| ENSG00000181104 | P00011 | Blood coagulation |
| ENSG00000137714 | P04396 | Vitamin D metabolism and pathway |
| ENSG00000129682 | P00021 | FGF signaling pathway |
| ENSG00000000938 | P00049 | Parkinson disease |
| ENSG00000187474 | P00031 | Inflammation mediated by chemokine and cytokine signaling pathway |
| ENSG00000010810 | P00007 | Axon guidance mediated by semaphorins |
| ENSG00000010810 | P00012 | Cadherin signaling pathway |
| ENSG00000010810 | P00034 | Integrin signalling pathway |
| ENSG00000010810 | P00049 | Parkinson disease |
| ENSG00000204681 | P05731 | GABA-B_receptor_II_signaling |
| ENSG00000206466 | P05731 | GABA-B_receptor_II_signaling |
| ENSG00000206511 | P05731 | GABA-B_receptor_II_signaling |
| ENSG00000232569 | P05731 | GABA-B_receptor_II_signaling |
| ENSG00000232632 | P05731 | GABA-B_receptor_II_signaling |
| ENSG00000237051 | P05731 | GABA-B_receptor_II_signaling |
| ENSG00000237112 | P05731 | GABA-B_receptor_II_signaling |
| ENSG00000154727 | P00047 | PDGF signaling pathway |
| ENSG00000258643 | P00006 | Apoptosis signaling pathway |
| ENSG00000132965 | P00031 | Inflammation mediated by chemokine and cytokine signaling pathway |
| ENSG00000112699 | P02752 | Mannose metabolism |
| ENSG00000167083 | P00026 | Heterotrimeric G-protein signaling pathway-Gi alpha and Gs alpha |
| ENSG00000167083 | P00027 | Heterotrimeric G-protein signaling pathway-Gq alpha and Go alpha |
| ENSG00000167083 | P00028 | Heterotrimeric G-protein signaling pathway-rod outer segment phototransduction |
| ENSG00000167083 | P00031 | Inflammation mediated by chemokine and cytokine signaling pathway |
| ENSG00000167083 | P00039 | Metabotropic glutamate receptor group III pathway |
| ENSG00000167083 | P00040 | Metabotropic glutamate receptor group II pathway |
| ENSG00000167083 | P00042 | Muscarinic acetylcholine receptor 1 and 3 signaling pathway |
| ENSG00000167083 | P00043 | Muscarinic acetylcholine receptor 2 and 4 signaling pathway |
| ENSG00000167083 | P00048 | PI3 kinase pathway |
| ENSG00000167083 | P00057 | Wnt signaling pathway |
| ENSG00000167083 | P04373 | 5HT1 type receptor mediated signaling pathway |
| ENSG00000167083 | P04374 | 5HT2 type receptor mediated signaling pathway |
| ENSG00000167083 | P04376 | 5HT4 type receptor mediated signaling pathway |
| ENSG00000167083 | P04377 | Beta1 adrenergic receptor signaling pathway |
| ENSG00000167083 | P04378 | Beta2 adrenergic receptor signaling pathway |
| ENSG00000167083 | P04379 | Beta3 adrenergic receptor signaling pathway |
| ENSG00000167083 | P04380 | Cortocotropin releasing factor receptor signaling pathway |
| ENSG00000167083 | P04385 | Histamine H1 receptor mediated signaling pathway |
| ENSG00000167083 | P04386 | Histamine H2 receptor mediated signaling pathway |
| ENSG00000167083 | P04391 | Oxytocin receptor mediated signaling pathway |
| ENSG00000167083 | P04394 | Thyrotropin-releasing hormone receptor signaling pathway |
| ENSG00000167083 | P05730 | Endogenous_cannabinoid_signaling |
| ENSG00000167083 | P05731 | GABA-B_receptor_II_signaling |
| ENSG00000167083 | P05911 | Angiotensin II-stimulated signaling through G proteins and beta-arrestin |
| ENSG00000167083 | P05913 | Enkephalin release |
| ENSG00000167083 | P05915 | Opioid proenkephalin pathway |
| ENSG00000167083 | P05916 | Opioid prodynorphin pathway |
| ENSG00000167083 | P05917 | Opioid proopiomelanocortin pathway |
| ENSG00000186810 | P00031 | Inflammation mediated by chemokine and cytokine signaling pathway |
| ENSG00000198055 | P00026 | Heterotrimeric G-protein signaling pathway-Gi alpha and Gs alpha |
| ENSG00000198055 | P00027 | Heterotrimeric G-protein signaling pathway-Gq alpha and Go alpha |
| ENSG00000198055 | P00031 | Inflammation mediated by chemokine and cytokine signaling pathway |
| ENSG00000198055 | P05911 | Angiotensin II-stimulated signaling through G proteins and beta-arrestin |
| ENSG00000177885 | P00005 | Angiogenesis |
| ENSG00000177885 | P00010 | B cell activation |
| ENSG00000177885 | P00018 | EGF receptor signaling pathway |
| ENSG00000177885 | P00021 | FGF signaling pathway |
| ENSG00000177885 | P00031 | Inflammation mediated by chemokine and cytokine signaling pathway |
| ENSG00000177885 | P00032 | Insulin/IGF pathway-mitogen activated protein kinase kinase/MAP kinase cascade |
| ENSG00000177885 | P00034 | Integrin signalling pathway |
| ENSG00000177885 | P00036 | Interleukin signaling pathway |
| ENSG00000177885 | P00047 | PDGF signaling pathway |
| ENSG00000177885 | P00048 | PI3 kinase pathway |
| ENSG00000177885 | P00053 | T cell activation |
| ENSG00000177885 | P04393 | Ras Pathway |
| ENSG00000177885 | P05912 | Dopamine receptor mediated signaling pathway |
| ENSG00000177885 | P06587 | Nicotine pharmacodynamics pathway |
| ENSG00000177885 | P06664 | Gonadotropin releasing hormone receptor pathway |
| ENSG00000177885 | P06959 | CCKR signaling map |
| ENSG00000164418 | P00029 | Huntington disease |
| ENSG00000164418 | P00037 | Ionotropic glutamate receptor pathway |
| ENSG00000164418 | P00039 | Metabotropic glutamate receptor group III pathway |
| ENSG00000179603 | P00026 | Heterotrimeric G-protein signaling pathway-Gi alpha and Gs alpha |
| ENSG00000179603 | P00027 | Heterotrimeric G-protein signaling pathway-Gq alpha and Go alpha |
| ENSG00000179603 | P00039 | Metabotropic glutamate receptor group III pathway |
| ENSG00000163739 | P06959 | CCKR signaling map |
| ENSG00000152402 | P00019 | Endothelin signaling pathway |
| ENSG00000143774 | P02738 | De novo purine biosynthesis |
| ENSG00000101336 | P00049 | Parkinson disease |
| ENSG00000168384 | P00053 | T cell activation |
| ENSG00000206291 | P00053 | T cell activation |
| ENSG00000224103 | P00053 | T cell activation |
| ENSG00000228163 | P00053 | T cell activation |
| ENSG00000229685 | P00053 | T cell activation |
| ENSG00000231389 | P00053 | T cell activation |
| ENSG00000235844 | P00053 | T cell activation |
| ENSG00000236177 | P00053 | T cell activation |
| ENSG00000196735 | P00053 | T cell activation |
| ENSG00000206305 | P00053 | T cell activation |
| ENSG00000225890 | P00053 | T cell activation |
| ENSG00000228284 | P00053 | T cell activation |
| ENSG00000232062 | P00053 | T cell activation |
| ENSG00000236418 | P00053 | T cell activation |
| ENSG00000204287 | P00053 | T cell activation |
| ENSG00000206308 | P00053 | T cell activation |
| ENSG00000226260 | P00053 | T cell activation |
| ENSG00000227993 | P00053 | T cell activation |
| ENSG00000228987 | P00053 | T cell activation |
| ENSG00000230726 | P00053 | T cell activation |
| ENSG00000234794 | P00053 | T cell activation |
| ENSG00000277263 | P00053 | T cell activation |
| ENSG00000189403 | P00059 | p53 pathway |
| ENSG00000086696 | P02727 | Androgen/estrogene/progesterone biosynthesis |
| ENSG00000211899 | P00010 | B cell activation |
| ENSG00000282657 | P00010 | B cell activation |
| ENSG00000110324 | P00036 | Interleukin signaling pathway |
| ENSG00000095752 | P00036 | Interleukin signaling pathway |
| ENSG00000081985 | P00036 | Interleukin signaling pathway |
| ENSG00000100385 | P00036 | Interleukin signaling pathway |
| ENSG00000163464 | P00031 | Inflammation mediated by chemokine and cytokine signaling pathway |
| ENSG00000163464 | P00036 | Interleukin signaling pathway |
| ENSG00000180871 | P00031 | Inflammation mediated by chemokine and cytokine signaling pathway |
| ENSG00000180871 | P00036 | Interleukin signaling pathway |
| ENSG00000171105 | P00032 | Insulin/IGF pathway-mitogen activated protein kinase kinase/MAP kinase cascade |
| ENSG00000171105 | P00033 | Insulin/IGF pathway-protein kinase B signaling cascade |
| ENSG00000171105 | P00048 | PI3 kinase pathway |
| ENSG00000171105 | P06664 | Gonadotropin releasing hormone receptor pathway |
| ENSG00000156886 | P00034 | Integrin signalling pathway |
| ENSG00000083457 | P00034 | Integrin signalling pathway |
| ENSG00000169896 | P00031 | Inflammation mediated by chemokine and cytokine signaling pathway |
| ENSG00000169896 | P00034 | Integrin signalling pathway |
| ENSG00000140678 | P00034 | Integrin signalling pathway |
| ENSG00000160255 | P00031 | Inflammation mediated by chemokine and cytokine signaling pathway |
| ENSG00000160255 | P00034 | Integrin signalling pathway |
| ENSG00000123104 | P00010 | B cell activation |
| ENSG00000123104 | P00019 | Endothelin signaling pathway |
| ENSG00000123104 | P00027 | Heterotrimeric G-protein signaling pathway-Gq alpha and Go alpha |
| ENSG00000123104 | P00031 | Inflammation mediated by chemokine and cytokine signaling pathway |
| ENSG00000123104 | P00042 | Muscarinic acetylcholine receptor 1 and 3 signaling pathway |
| ENSG00000123104 | P00047 | PDGF signaling pathway |
| ENSG00000123104 | P00057 | Wnt signaling pathway |
| ENSG00000123104 | P04385 | Histamine H1 receptor mediated signaling pathway |
| ENSG00000123104 | P05911 | Angiotensin II-stimulated signaling through G proteins and beta-arrestin |
| ENSG00000123104 | P06664 | Gonadotropin releasing hormone receptor pathway |
| ENSG00000101384 | P00005 | Angiogenesis |
| ENSG00000101384 | P00045 | Notch signaling pathway |
| ENSG00000120457 | P00026 | Heterotrimeric G-protein signaling pathway-Gi alpha and Gs alpha |
| ENSG00000120457 | P00027 | Heterotrimeric G-protein signaling pathway-Gq alpha and Go alpha |
| ENSG00000120457 | P00043 | Muscarinic acetylcholine receptor 2 and 4 signaling pathway |
| ENSG00000143761 | P00029 | Huntington disease |
| ENSG00000143761 | P00034 | Integrin signalling pathway |
| ENSG00000043462 | P00053 | T cell activation |
| ENSG00000165527 | P00029 | Huntington disease |
| ENSG00000165527 | P00034 | Integrin signalling pathway |
| ENSG00000107798 | P02727 | Androgen/estrogene/progesterone biosynthesis |
| ENSG00000079435 | P02782 | Triacylglycerol metabolism |
| ENSG00000155366 | P00005 | Angiogenesis |
| ENSG00000155366 | P00007 | Axon guidance mediated by semaphorins |
| ENSG00000155366 | P00008 | Axon guidance mediated by Slit/Robo |
| ENSG00000155366 | P00016 | Cytoskeletal regulation by Rho GTPase |
| ENSG00000155366 | P00027 | Heterotrimeric G-protein signaling pathway-Gq alpha and Go alpha |
| ENSG00000155366 | P00031 | Inflammation mediated by chemokine and cytokine signaling pathway |
| ENSG00000155366 | P00034 | Integrin signalling pathway |
| ENSG00000155366 | P04393 | Ras Pathway |
| ENSG00000155366 | P05911 | Angiotensin II-stimulated signaling through G proteins and beta-arrestin |
| ENSG00000070018 | P00004 | Alzheimer disease-presenilin pathway |
| ENSG00000070018 | P00057 | Wnt signaling pathway |
| ENSG00000281324 | P00004 | Alzheimer disease-presenilin pathway |
| ENSG00000281324 | P00057 | Wnt signaling pathway |
| ENSG00000204487 | P00006 | Apoptosis signaling pathway |
| ENSG00000206437 | P00006 | Apoptosis signaling pathway |
| ENSG00000223448 | P00006 | Apoptosis signaling pathway |
| ENSG00000227507 | P00006 | Apoptosis signaling pathway |
| ENSG00000231314 | P00006 | Apoptosis signaling pathway |
| ENSG00000236237 | P00006 | Apoptosis signaling pathway |
| ENSG00000236925 | P00006 | Apoptosis signaling pathway |
| ENSG00000238114 | P00006 | Apoptosis signaling pathway |
| ENSG00000137834 | P00052 | TGF-beta signaling pathway |
| ENSG00000006062 | P00006 | Apoptosis signaling pathway |
| ENSG00000006062 | P00018 | EGF receptor signaling pathway |
| ENSG00000006062 | P06664 | Gonadotropin releasing hormone receptor pathway |
| ENSG00000006062 | P06959 | CCKR signaling map |
| ENSG00000282637 | P00006 | Apoptosis signaling pathway |
| ENSG00000282637 | P00018 | EGF receptor signaling pathway |
| ENSG00000282637 | P06664 | Gonadotropin releasing hormone receptor pathway |
| ENSG00000282637 | P06959 | CCKR signaling map |
| ENSG00000198909 | P00010 | B cell activation |
| ENSG00000198909 | P00018 | EGF receptor signaling pathway |
| ENSG00000198909 | P00021 | FGF signaling pathway |
| ENSG00000198909 | P00034 | Integrin signalling pathway |
| ENSG00000198909 | P06664 | Gonadotropin releasing hormone receptor pathway |
| ENSG00000104814 | P00006 | Apoptosis signaling pathway |
| ENSG00000104814 | P06664 | Gonadotropin releasing hormone receptor pathway |
| ENSG00000282928 | P00006 | Apoptosis signaling pathway |
| ENSG00000282928 | P06664 | Gonadotropin releasing hormone receptor pathway |
| ENSG00000198625 | P00033 | Insulin/IGF pathway-protein kinase B signaling cascade |
| ENSG00000198625 | P00059 | p53 pathway |
| ENSG00000198625 | P04392 | P53 pathway feedback loops 1 |
| ENSG00000198625 | P04398 | p53 pathway feedback loops 2 |
| ENSG00000137486 | P00026 | Heterotrimeric G-protein signaling pathway-Gi alpha and Gs alpha |
| ENSG00000137486 | P00031 | Inflammation mediated by chemokine and cytokine signaling pathway |
| ENSG00000137486 | P00057 | Wnt signaling pathway |
| ENSG00000137486 | P05911 | Angiotensin II-stimulated signaling through G proteins and beta-arrestin |
| ENSG00000141480 | P00026 | Heterotrimeric G-protein signaling pathway-Gi alpha and Gs alpha |
| ENSG00000141480 | P00031 | Inflammation mediated by chemokine and cytokine signaling pathway |
| ENSG00000141480 | P00057 | Wnt signaling pathway |
| ENSG00000141480 | P05911 | Angiotensin II-stimulated signaling through G proteins and beta-arrestin |
| ENSG00000141480 | P06959 | CCKR signaling map |
| ENSG00000215914 | P00004 | Alzheimer disease-presenilin pathway |
| ENSG00000167508 | P00014 | Cholesterol biosynthesis |
| ENSG00000070669 | P02730 | Asparagine and aspartate biosynthesis |
| ENSG00000091536 | P00044 | Nicotinic acetylcholine receptor signaling pathway |
| ENSG00000128641 | P00044 | Nicotinic acetylcholine receptor signaling pathway |
| ENSG00000176658 | P00044 | Nicotinic acetylcholine receptor signaling pathway |
| ENSG00000142347 | P00044 | Nicotinic acetylcholine receptor signaling pathway |
| ENSG00000197535 | P00044 | Nicotinic acetylcholine receptor signaling pathway |
| ENSG00000169994 | P00044 | Nicotinic acetylcholine receptor signaling pathway |
| ENSG00000099331 | P00044 | Nicotinic acetylcholine receptor signaling pathway |
| ENSG00000107954 | P00045 | Notch signaling pathway |
| ENSG00000196712 | P00018 | EGF receptor signaling pathway |
| ENSG00000101096 | P00009 | Axon guidance mediated by netrin |
| ENSG00000101096 | P00010 | B cell activation |
| ENSG00000101096 | P00031 | Inflammation mediated by chemokine and cytokine signaling pathway |
| ENSG00000101096 | P00053 | T cell activation |
| ENSG00000101096 | P00057 | Wnt signaling pathway |
| ENSG00000101096 | P06664 | Gonadotropin releasing hormone receptor pathway |
| ENSG00000101096 | P06959 | CCKR signaling map |
| ENSG00000007171 | P00048 | PI3 kinase pathway |
| ENSG00000099250 | P00007 | Axon guidance mediated by semaphorins |
| ENSG00000133961 | P00045 | Notch signaling pathway |
| ENSG00000125510 | P00026 | Heterotrimeric G-protein signaling pathway-Gi alpha and Gs alpha |
| ENSG00000125510 | P00027 | Heterotrimeric G-protein signaling pathway-Gq alpha and Go alpha |
| ENSG00000277044 | P00026 | Heterotrimeric G-protein signaling pathway-Gi alpha and Gs alpha |
| ENSG00000277044 | P00027 | Heterotrimeric G-protein signaling pathway-Gq alpha and Go alpha |
| ENSG00000124507 | P00029 | Huntington disease |
| ENSG00000113555 | P00012 | Cadherin signaling pathway |
| ENSG00000113555 | P00057 | Wnt signaling pathway |
| ENSG00000255408 | P00012 | Cadherin signaling pathway |
| ENSG00000255408 | P00057 | Wnt signaling pathway |
| ENSG00000204967 | P00012 | Cadherin signaling pathway |
| ENSG00000204967 | P00057 | Wnt signaling pathway |
| ENSG00000204965 | P00012 | Cadherin signaling pathway |
| ENSG00000204965 | P00057 | Wnt signaling pathway |
| ENSG00000204963 | P00012 | Cadherin signaling pathway |
| ENSG00000204963 | P00057 | Wnt signaling pathway |
| ENSG00000187372 | P00012 | Cadherin signaling pathway |
| ENSG00000187372 | P00057 | Wnt signaling pathway |
| ENSG00000120327 | P00012 | Cadherin signaling pathway |
| ENSG00000120327 | P00057 | Wnt signaling pathway |
| ENSG00000113212 | P00012 | Cadherin signaling pathway |
| ENSG00000113212 | P00057 | Wnt signaling pathway |
| ENSG00000253846 | P00012 | Cadherin signaling pathway |
| ENSG00000253846 | P00057 | Wnt signaling pathway |
| ENSG00000253767 | P00012 | Cadherin signaling pathway |
| ENSG00000253767 | P00057 | Wnt signaling pathway |
| ENSG00000185527 | P00028 | Heterotrimeric G-protein signaling pathway-rod outer segment phototransduction |
| ENSG00000141959 | P00024 | Glycolysis |
| ENSG00000171608 | P00005 | Angiogenesis |
| ENSG00000171608 | P00006 | Apoptosis signaling pathway |
| ENSG00000171608 | P00009 | Axon guidance mediated by netrin |
| ENSG00000171608 | P00010 | B cell activation |
| ENSG00000171608 | P00018 | EGF receptor signaling pathway |
| ENSG00000171608 | P00019 | Endothelin signaling pathway |
| ENSG00000171608 | P00021 | FGF signaling pathway |
| ENSG00000171608 | P00030 | Hypoxia response via HIF activation |
| ENSG00000171608 | P00031 | Inflammation mediated by chemokine and cytokine signaling pathway |
| ENSG00000171608 | P00033 | Insulin/IGF pathway-protein kinase B signaling cascade |
| ENSG00000171608 | P00034 | Integrin signalling pathway |
| ENSG00000171608 | P00047 | PDGF signaling pathway |
| ENSG00000171608 | P00053 | T cell activation |
| ENSG00000171608 | P00056 | VEGF signaling pathway |
| ENSG00000171608 | P00059 | p53 pathway |
| ENSG00000171608 | P04393 | Ras Pathway |
| ENSG00000171608 | P04398 | p53 pathway feedback loops 2 |
| ENSG00000105499 | P00005 | Angiogenesis |
| ENSG00000105499 | P06664 | Gonadotropin releasing hormone receptor pathway |
| ENSG00000101333 | P00002 | Alpha adrenergic receptor signaling pathway |
| ENSG00000101333 | P00019 | Endothelin signaling pathway |
| ENSG00000101333 | P00027 | Heterotrimeric G-protein signaling pathway-Gq alpha and Go alpha |
| ENSG00000101333 | P00031 | Inflammation mediated by chemokine and cytokine signaling pathway |
| ENSG00000101333 | P00041 | Metabotropic glutamate receptor group I pathway |
| ENSG00000101333 | P00042 | Muscarinic acetylcholine receptor 1 and 3 signaling pathway |
| ENSG00000101333 | P00057 | Wnt signaling pathway |
| ENSG00000101333 | P04374 | 5HT2 type receptor mediated signaling pathway |
| ENSG00000101333 | P04385 | Histamine H1 receptor mediated signaling pathway |
| ENSG00000101333 | P04391 | Oxytocin receptor mediated signaling pathway |
| ENSG00000101333 | P04394 | Thyrotropin-releasing hormone receptor signaling pathway |
| ENSG00000108387 | P00049 | Parkinson disease |
| ENSG00000120910 | P00010 | B cell activation |
| ENSG00000120910 | P00053 | T cell activation |
| ENSG00000120910 | P00057 | Wnt signaling pathway |
| ENSG00000163932 | P00002 | Alpha adrenergic receptor signaling pathway |
| ENSG00000163932 | P00003 | Alzheimer disease-amyloid secretase pathway |
| ENSG00000163932 | P00005 | Angiogenesis |
| ENSG00000163932 | P00006 | Apoptosis signaling pathway |
| ENSG00000163932 | P00010 | B cell activation |
| ENSG00000163932 | P00018 | EGF receptor signaling pathway |
| ENSG00000163932 | P00019 | Endothelin signaling pathway |
| ENSG00000163932 | P00021 | FGF signaling pathway |
| ENSG00000163932 | P00027 | Heterotrimeric G-protein signaling pathway-Gq alpha and Go alpha |
| ENSG00000163932 | P00042 | Muscarinic acetylcholine receptor 1 and 3 signaling pathway |
| ENSG00000163932 | P00056 | VEGF signaling pathway |
| ENSG00000163932 | P00057 | Wnt signaling pathway |
| ENSG00000163932 | P04374 | 5HT2 type receptor mediated signaling pathway |
| ENSG00000163932 | P04385 | Histamine H1 receptor mediated signaling pathway |
| ENSG00000163932 | P04391 | Oxytocin receptor mediated signaling pathway |
| ENSG00000163932 | P04394 | Thyrotropin-releasing hormone receptor signaling pathway |
| ENSG00000163932 | P06664 | Gonadotropin releasing hormone receptor pathway |
| ENSG00000163932 | P06959 | CCKR signaling map |
| ENSG00000181790 | P00059 | p53 pathway |
| ENSG00000101182 | P00049 | Parkinson disease |
| ENSG00000120899 | P00031 | Inflammation mediated by chemokine and cytokine signaling pathway |
| ENSG00000120899 | P00034 | Integrin signalling pathway |
| ENSG00000120899 | P06664 | Gonadotropin releasing hormone receptor pathway |
| ENSG00000120899 | P06959 | CCKR signaling map |
| ENSG00000111679 | P00005 | Angiogenesis |
| ENSG00000111679 | P00010 | B cell activation |
| ENSG00000111679 | P00021 | FGF signaling pathway |
| ENSG00000111679 | P00035 | Interferon-gamma signaling pathway |
| ENSG00000081237 | P00010 | B cell activation |
| ENSG00000081237 | P00038 | JAK/STAT signaling pathway |
| ENSG00000081237 | P00053 | T cell activation |
| ENSG00000262418 | P00010 | B cell activation |
| ENSG00000262418 | P00038 | JAK/STAT signaling pathway |
| ENSG00000262418 | P00053 | T cell activation |
| ENSG00000128340 | P00007 | Axon guidance mediated by semaphorins |
| ENSG00000128340 | P00008 | Axon guidance mediated by Slit/Robo |
| ENSG00000128340 | P00009 | Axon guidance mediated by netrin |
| ENSG00000128340 | P00010 | B cell activation |
| ENSG00000128340 | P00016 | Cytoskeletal regulation by Rho GTPase |
| ENSG00000128340 | P00018 | EGF receptor signaling pathway |
| ENSG00000128340 | P00021 | FGF signaling pathway |
| ENSG00000128340 | P00029 | Huntington disease |
| ENSG00000128340 | P00031 | Inflammation mediated by chemokine and cytokine signaling pathway |
| ENSG00000128340 | P00034 | Integrin signalling pathway |
| ENSG00000128340 | P00053 | T cell activation |
| ENSG00000128340 | P00056 | VEGF signaling pathway |
| ENSG00000128340 | P04393 | Ras Pathway |
| ENSG00000128340 | P05918 | p38 MAPK pathway |
| ENSG00000172575 | P00027 | Heterotrimeric G-protein signaling pathway-Gq alpha and Go alpha |
| ENSG00000171791 | P00006 | Apoptosis signaling pathway |
| ENSG00000171791 | P00046 | Oxidative stress response |
| ENSG00000171791 | P06959 | CCKR signaling map |
| ENSG00000090104 | P00026 | Heterotrimeric G-protein signaling pathway-Gi alpha and Gs alpha |
| ENSG00000090104 | P00027 | Heterotrimeric G-protein signaling pathway-Gq alpha and Go alpha |
| ENSG00000090104 | P00031 | Inflammation mediated by chemokine and cytokine signaling pathway |
| ENSG00000127074 | P00026 | Heterotrimeric G-protein signaling pathway-Gi alpha and Gs alpha |
| ENSG00000127074 | P00027 | Heterotrimeric G-protein signaling pathway-Gq alpha and Go alpha |
| ENSG00000127074 | P00031 | Inflammation mediated by chemokine and cytokine signaling pathway |
| **5-Fluorouracil Gene List** | |  |
| **Ensemble gene ID** | **Pathway ID** | **Pathway Name** |
| ENSG00000187122 | P00008 | Axon guidance mediated by Slit/Robo |
| ENSG00000153147 | P00057 | Wnt signaling pathway |
| ENSG00000139613 | P00057 | Wnt signaling pathway |
| ENSG00000115904 | P00005 | Angiogenesis |
| ENSG00000115904 | P00010 | B cell activation |
| ENSG00000115904 | P00018 | EGF receptor signaling pathway |
| ENSG00000115904 | P00021 | FGF signaling pathway |
| ENSG00000115904 | P00031 | Inflammation mediated by chemokine and |
| ENSG00000115904 | P00032 | Insulin/IGF pathway-mitogen activated protein kinase |
| ENSG00000115904 | P00034 | Integrin signalling pathway |
| ENSG00000115904 | P00036 | Interleukin signaling pathway |
| ENSG00000115904 | P00047 | PDGF signaling pathway |
| ENSG00000115904 | P00048 | PI3 kinase pathway |
| ENSG00000115904 | P00053 | T cell activation |
| ENSG00000115904 | P04393 | Ras Pathway |
| ENSG00000115904 | P06664 | Gonadotropin releasing hormone receptor pathway |
| ENSG00000115904 | P06959 | CCKR signaling map |
| ENSG00000070808 | P00031 | Inflammation mediated by chemokine and |
| ENSG00000070808 | P00037 | Ionotropic glutamate receptor pathway |
| ENSG00000110395 | P00018 | EGF receptor signaling pathway |
| ENSG00000101199 | P00034 | Integrin signalling pathway |
| ENSG00000078814 | P00016 | Cytoskeletal regulation by Rho GTPase |
| ENSG00000078814 | P00031 | Inflammation mediated by chemokine and |
| ENSG00000078814 | P00044 | Nicotinic acetylcholine receptor signaling pathway |
| ENSG00000078814 | P00057 | Wnt signaling pathway |
| ENSG00000198276 | P02775 | Salvage pyrimidine ribonucleotides |
| ENSG00000009335 | P00060 | Ubiquitin proteasome pathway |
| ENSG00000145819 | P00034 | Integrin signalling pathway |
| ENSG00000145819 | P00047 | PDGF signaling pathway |
| ENSG00000156650 | P00059 | p53 pathway |
| ENSG00000281813 | P00059 | p53 pathway |
| ENSG00000049618 | P00057 | Wnt signaling pathway |
| ENSG00000172602 | P00034 | Integrin signalling pathway |
| ENSG00000168615 | P00003 | Alzheimer disease-amyloid secretase pathway |
| ENSG00000282230 | P00003 | Alzheimer disease-amyloid secretase pathway |
| ENSG00000033800 | P00035 | Interferon-gamma signaling pathway |
| ENSG00000033800 | P00038 | JAK/STAT signaling pathway |
| ENSG00000004975 | P00004 | Alzheimer disease-presenilin pathway |
| ENSG00000004975 | P00005 | Angiogenesis |
| ENSG00000004975 | P00057 | Wnt signaling pathway |
| ENSG00000100393 | P00026 | Heterotrimeric G-protein signaling pathway-Gi alpha |
| ENSG00000100393 | P00029 | Huntington disease |
| ENSG00000100393 | P00052 | TGF-beta signaling pathway |
| ENSG00000100393 | P00055 | Transcription regulation by bZIP transcription |
| ENSG00000100393 | P00057 | Wnt signaling pathway |
| ENSG00000100393 | P00059 | p53 pathway |
| ENSG00000100393 | P06211 | BMP_signaling_pathway-drosophila |
| ENSG00000100393 | P06212 | DPP-SCW_signaling_pathway |
| ENSG00000100393 | P06213 | DPP_signaling_pathway |
| ENSG00000100393 | P06214 | GBB_signaling_pathway |
| ENSG00000100393 | P06216 | SCW_signaling_pathway |
| ENSG00000100393 | P06664 | Gonadotropin releasing hormone receptor pathway |
| ENSG00000151422 | P00012 | Cadherin signaling pathway |
| ENSG00000213930 | P02744 | Fructose galactose metabolism |
| ENSG00000161905 | P00031 | Inflammation mediated by chemokine and |
| ENSG00000161905 | P06664 | Gonadotropin releasing hormone receptor pathway |
| ENSG00000105464 | P00029 | Huntington disease |
| ENSG00000105464 | P00037 | Ionotropic glutamate receptor pathway |
| ENSG00000105464 | P00039 | Metabotropic glutamate receptor group III |
| ENSG00000105464 | P00041 | Metabotropic glutamate receptor group I |
| ENSG00000105464 | P00042 | Muscarinic acetylcholine receptor 1 and |
| ENSG00000197386 | P00029 | Huntington disease |
| ENSG00000173110 | P00006 | Apoptosis signaling pathway |
| ENSG00000173110 | P00049 | Parkinson disease |
| ENSG00000120868 | P00006 | Apoptosis signaling pathway |
| ENSG00000120868 | P00020 | FAS signaling pathway |
| ENSG00000120868 | P00029 | Huntington disease |
| ENSG00000120868 | P00059 | p53 pathway |
| ENSG00000185507 | P00054 | Toll receptor signaling pathway |
| ENSG00000276561 | P00054 | Toll receptor signaling pathway |
| ENSG00000169967 | P00010 | B cell activation |
| ENSG00000169967 | P00018 | EGF receptor signaling pathway |
| ENSG00000169967 | P00021 | FGF signaling pathway |
| ENSG00000169967 | P00034 | Integrin signalling pathway |
| ENSG00000169967 | P00047 | PDGF signaling pathway |
| ENSG00000169967 | P06664 | Gonadotropin releasing hormone receptor pathway |
| ENSG00000120500 | P00057 | Wnt signaling pathway |
| ENSG00000066136 | P06664 | Gonadotropin releasing hormone receptor pathway |
| ENSG00000177463 | P06959 | CCKR signaling map |
| ENSG00000138801 | P02778 | Sulfate assimilation |
| ENSG00000204967 | P00012 | Cadherin signaling pathway |
| ENSG00000204967 | P00057 | Wnt signaling pathway |
| ENSG00000253953 | P00012 | Cadherin signaling pathway |
| ENSG00000253953 | P00057 | Wnt signaling pathway |
| ENSG00000112033 | P00057 | Wnt signaling pathway |
| ENSG00000050820 | P00034 | Integrin signalling pathway |
| ENSG00000050820 | P06959 | CCKR signaling map |
| ENSG00000285460 | P00034 | Integrin signalling pathway |
| ENSG00000285460 | P06959 | CCKR signaling map |
| ENSG00000035928 | P00017 | DNA replication |

# **S2 Table: Top 20 PANTHER pathways in models by gene percent.**

| **Model** | | **Category name** | **Accession** | **# Genes** | **Percent of gene hit against total # genes** | **Percent of gene hit against total # pathway hit** | **raw P Value** | **FDR** |
| --- | --- | --- | --- | --- | --- | --- | --- | --- |
| 5-Flourouracil | Wnt signaling pathway | | P00057 | 10 | 3.20% | 11.80% | 2.82E-02 | 4.60E+00 |
|  | Integrin signaling pathway | | P00034 | 6 | 1.90% | 7.10% | 6.60E-02 | 3.59E+00 |
|  | Gonadotropin-releasing hormone receptor pathway | | P06664 | 5 | 1.60% | 5.90% | 4.06E-01 | 2.88E+00 |
|  | Inflammation mediated by chemokine and cytokine signaling pathway | | P00031 | 4 | 1.30% | 4.70% | 7.91E-01 | 3.39E+00 |
|  | Huntington disease | | P00029 | 4 | 1.30% | 4.70% | 1.70E-01 | 2.78E+00 |
|  | p53 pathway | | P00059 | 3 | 1.00% | 3.50% | 1.49E-01 | 2.69E+00 |
|  | EGF receptor signaling pathway | | P00018 | 3 | 1.00% | 3.50% | 4.66E-01 | 3.16E+00 |
|  | PDGF signaling pathway | | P00047 | 3 | 1.00% | 3.50% | 4.88E-01 | 3.18E+00 |
|  | Cadherin signaling pathway | | P00012 | 3 | 1.00% | 3.50% | 5.08E-01 | 3.18E+00 |
|  | CCKR signaling map | | P06959 | 3 | 1.00% | 3.50% | 7.45E-01 | 3.28E+00 |
|  | Apoptosis signaling pathway | | P00006 | 2 | 0.60% | 2.40% | 6.97E-01 | 3.34E+00 |
|  | Angiogenesis | | P00005 | 2 | 0.60% | 2.40% | 1.00E+00 | 1.00E+00 |
|  | Ionotropic glutamate receptor pathway | | P00037 | 2 | 0.60% | 2.40% | 1.74E-01 | 2.36E+00 |
|  | FGF signaling pathway | | P00021 | 2 | 0.60% | 2.40% | 7.03E-01 | 3.27E+00 |
|  | B cell activation | | P00010 | 2 | 0.60% | 2.40% | 2.74E-01 | 2.80E+00 |
|  | DPP signaling pathway | | P06213 | 1 | 0.30% | 1.20% | 1.10E-01 | 2.56E+00 |
|  | DPP-SCW signaling pathway | | P06212 | 1 | 0.30% | 1.20% | 9.70E-02 | 2.64E+00 |
|  | BMP/activin signaling pathway-drosophila | | P06211 | 1 | 0.30% | 1.20% | 1.10E-01 | 2.24E+00 |
|  | Axon guidance mediated by Slit/Robo | | P00008 | 1 | 0.30% | 1.20% | 1.10E-01 | 2.24E+00 |
|  | Metabotropic glutamate receptor group III pathway | | P00039 | 1 | 0.30% | 1.20% | 1.10E-01 | 2.24E+00 |
| Gemcitabine | Inflammation mediated by chemokine and cytokine signaling pathway | | P00031 | 33 | 2.80% | 6.70% | 2.28E-05 | 9.27E-04 |
|  | Wnt signaling pathway | | P00057 | 28 | 2.40% | 5.70% | 1.85E-02 | 3.02E-01 |
|  | T cell activation | | P00053 | 21 | 1.80% | 4.30% | 2.95E-07 | 2.40E-05 |
|  | Heterotrimeric G-protein signaling pathway-Gq alpha and Go alpha mediated pathway | | P00027 | 17 | 1.50% | 3.50% | 1.74E-03 | 5.68E-02 |
|  | B cell activation | | P00010 | 17 | 1.50% | 3.50% | 1.82E-06 | 9.91E-05 |
|  | Integrin signaling pathway | | P00034 | 16 | 1.40% | 3.30% | 1.12E-01 | 8.33E-01 |
|  | Angiogenesis | | P00005 | 15 | 1.30% | 3.10% | 9.70E-02 | 7.91E-01 |
|  | Heterotrimeric G-protein signaling pathway-Gi alpha and Gs alpha mediated pathway | | P00026 | 15 | 1.30% | 3.10% | 6.25E-02 | 7.27E-01 |
|  | Cadherin signaling pathway | | P00012 | 15 | 1.30% | 3.10% | 5.56E-02 | 6.97E-01 |
|  | Nicotinic acetylcholine receptor signaling pathway | | P00044 | 14 | 1.20% | 2.90% | 2.66E-03 | 7.22E-02 |
|  | Gonadotropin-releasing hormone receptor pathway | | P06664 | 13 | 1.10% | 2.70% | 1.00E+00 | 1.01E+00 |
|  | EGF receptor signaling pathway | | P00018 | 13 | 1.10% | 2.70% | 9.59E-02 | 8.22E-01 |
|  | Apoptosis signaling pathway | | P00006 | 10 | 0.90% | 2.00% | 2.29E-01 | 1.24E+00 |
|  | CCKR signaling map | | P06959 | 11 | 0.90% | 2.20% | 6.17E-01 | 1.60E+00 |
|  | Parkinson disease | | P00049 | 10 | 0.90% | 2.00% | 8.77E-02 | 8.41E-01 |
|  | Interleukin signaling pathway | | P00036 | 11 | 0.90% | 2.20% | 1.98E-02 | 2.93E-01 |
|  | PDGF signaling pathway | | P00047 | 9 | 0.80% | 1.80% | 7.21E-01 | 1.53E+00 |
|  | FGF signaling pathway | | P00021 | 9 | 0.80% | 1.80% | 4.27E-01 | 1.42E+00 |
|  | Huntington disease | | P00029 | 8 | 0.70% | 1.60% | 1.00E+00 | 1.00E+00 |

# **S3 Table: Accuracy by cancer type**

| **5-Fluorouracil Cancer Type** | **Accuracy** | **Count** |  |
| --- | --- | --- | --- |
| Colon adenocarcinoma | 85.71% | 7 |  |
| Esophageal carcinoma | 25.0% | 4 |  |
| Pancreatic adenocarcinoma | 90.0% | 10 |  |
| Rectum adenocarcinoma | 100.0% | 16 |  |
| Stomach adenocarcinoma | 76.0% | 25 |  |
| **Gemcitabine Cancer Type** | **Accuracy** | **Count** |  |
| Bladder Urothelial Carcinoma | 100.00% | 12 | |
| Breast invasive carcinoma | 100.0% | 3 | |
| Cervical squamous cell carcinoma and endocervical adenocarcinoma | 100.0% | 2 | |
| Cholangiocarcinoma | 100.0% | 7 | |
| Head and Neck squamous cell carcinoma | 100.0% | 2 | |
| Liver hepatocellular carcinoma | 100.0% | 3 | |
| Lung adenocarcinoma | 100.0% | 5 | |
| Ovarian serous cystadenocarcinom | 75.0% | 4 | |
| Pancreatic adenocarcinoma | 86.0% | 43 | |
| Pheochromocytoma and Paraganglioma | 100.0% | 2 | |
| Sarcoma | 88.9% | 9 | |
| Skin Cutaneous Melanoma | 100.0% | 2 | |
| Testicular Germ Cell Tumors | 100.0% | 1 | |
| Uterine Corpus Endometrial Carcinoma | 100.0% | 1 | |

# **Supplementary Methods: Data pre-processing, gene standardization and gene selection**

**Data Pre-processing**

Pre-processing was required to make the data viable for our study. The clinical data was first cleaned, and records were removed if vital data was missing. Records missing drug name, response, cancer type, days on drug therapy, or days to drug therapy start were removed. We also removed records for which gene expression data was not available. We required a sample size of at least 30 records for a drug to be considered for the study and at least 15 records for each response type.

**Gene Standardization & Gene Selection**

We used the Upper-Quartile normalized Fragments Per Kilobase of transcript per Million mapped reads (UQ-FPKM) files from TCGA. Each file contained approximately, 60,000 ensemble gene ids. To account for the differences in this gene expression data between cancer types, we calculated the mean (cancer type mean) and standard deviation (cancer type sd) of the logged expression values for every TCGA patient with a given cancer type. We standardized the gene expression for the patients included in the study by subtracting the cancer type mean and dividing by the cancer type standard deviation.

We reduced the number of genes to effectively use clustering algorithms. We wanted to retain the genes that have the highest amount of variation (standard deviation) and the highest number of unique values (S1 Fig). Genes with less than 70% unique values across all patients were removed first. Next, we calculated the standard deviation for each remaining gene. We used the product of the standard deviation and percentage of unique values to rank the remaining genes. The top 5,000 genes ranked by this method were included in the clustering algorithm, which is discussed in the next section. Genes selected by the variable selection method can be found in S1 Table.

**SPD Results**

We compared single cancer and pan-cancer models using Sample Progression Discovery (SPD) clustering and random forest classification. SPD was borrowed from a previously published paper, because it discovers patterns of biological progression within data by building relatively same-size clusters of arbitrary shape. Under SPD, the model cross-validation accuracy was 71.4% for 5-FU and 73.5% for GCB. The number of clusters created by SPD was higher for 5-FU (251) than GCB (202), but the number of clusters selected with random forest was lower (25 vs 35). The results of the single cancer models showed lower accuracy rates than the pan models: 5-FU STAD (70.90%) and GCB PAAD (67.7%). This result supported our decision to only focus on pan-cancer models for the remaining analysis of the study. For due diligence, we compared the results of a model with variables as gene clusters versus that of single genes. We looked at both when all data was used versus the top 10 most informative features. Informative features were determined using the mean Gini index. We found that when clustering was done, the model performed better. See results below:

- 1. Gene Cluster Accuracy
     1. Random Forest (All variables): 65.2%
     2. Random Forest (Top 10 variables): 72.5%
  2. Single Gene Accuracy
     1. Random Forest (All variables): 60.9%
     2. Random Forest (Top 10 variables): 70.0%

**Model Validation Set Investigation**

The limited size of the data set was also a bottleneck to performing a robust model validation. We tried two different validation methods. For the first method, we attempted to use the patients who had been treated with 5-FU or GCB but were missing a documented measure of response to treatment. We first proved that the survival data is a good predictor of drug response, and we were going to use survival curves to validate our predictions (See S2 Figure for additional information). Despite its promise, there were too few patients for this approach to be of value. As a second attempt, we used patients who were on combination therapy with the drugs of interest [5-FU/Leucovorin and Gemcitabine/Cisplatin,] as their first line of treatment. We performed the standard process for model validation (using all training data to train the model, and then testing accuracy on the testing data). The results were not promising [5-FU/Leucovorin: 53.1% and Gemcitabine/Cisplatin: 51.0%]. Thus, we left out half of the most populous cancer from the training set as described in the methods.
